# Gender Moderates the Influence of Self-Construal Priming on Fairness Considerations

**DOI:** 10.3389/fpsyg.2017.00503

**Published:** 2017-04-03

**Authors:** Nic Flinkenflogel, Sheida Novin, Mariette Huizinga, Lydia Krabbendam

**Affiliations:** ^1^VICI Lab, Department of Clinical, Neuro- and Developmental Psychology, VU UniversityAmsterdam, Netherlands; ^2^Department of Psychology, Utrecht UniversityUtrecht, Netherlands

**Keywords:** gender, self-construal, priming, culture, decision-making, ultimatum game

## Abstract

Research in social and cultural psychology has identified that self-construal, or the way the self is defined in relation to others, plays an important role in social decision-making processes. Yet it remains difficult to isolate the effect of self-construal in a comparative approach. Therefore, we used priming methodology in three studies to induce either an independent or interdependent mindset to test direct consequences on fairness considerations. Specifically, we asked whether participants would accept an unfair ultimatum game offer: a split of 10 euros, where the participant is allocated the marginal share of 3 and the proposer 7. If the participant refuses, neither gets paid. In the first study, we used the well-known similarities and differences prime. Here, activating an interdependent mindset decreased rejection of the unfair offer compared to the independent mindset and control condition, but only in females. The prime did not affect males. In the second and third study we modified our university's mission statement to instead include either independent or interdependent values. Females displayed a similar direction of effects; in males however, activating an interdependent mindset increased rejection. Taken together, the results show that whether participants accept or reject an unfair offer depends on both their gender and the self-construal prime. The results were interpreted using the distinction between relational independence that has been associated with females, and collective interdependence, that has been associated with males. Possible consequences for future studies are discussed.

## Introduction

Human social behavior does not follow the rational principles of economic models. Rather than maximizing individual profit, decision-making is driven by social preferences like inequality aversion, fairness, trust, and reciprocity (Weber et al., 2004; Balliet et al., 2009; Van Lange et al., 2013). Even when people are given the option to divide a sum between themselves and a different person without any possible repercussions or loss of face, the mode of behavior is generally a fair division (Hoffman et al., 1996; Diekmann, 2004). In addition, there is marked variation both within and between individuals, indicating that decision-making in social situations does not adhere to universal principles. Part of this variation can be explained by individual attributes such as gender and age (MacPherson et al., 2002; Deakin et al., 2004; Peters et al., 2007; Eckel and Grossman, 2008; Croson and Gneezy, 2009; Balliet et al., 2011), while a substantial amount also originates in the group the individual belongs to Oosterbeek et al. (2004), Wong and Hong (2005) and Gächter et al. (2010). However, it has proven difficult to disentangle the psychological mechanisms underlying differences in decision-making behavior (Grossmann and Na, 2014). Here we focus on self-construal as a plausible underlying psychological mechanism, and use priming in three experimental studies to further elucidate variation in the way people value outcomes for self and others.

Self-construal refers to the way the self is perceived and defined in relation to others. Cross-cultural studies demonstrate that self-construals differ between cultural groups. In predominantly individualistic cultures, as for example seen in Western European countries and North America, people typically perceive themselves as unique and independent from others (Markus and Kitayama, 1991; Triandis, 1993). The self is defined mainly in terms of internal attributes such as abilities and attitudes (Markus and Kitayama, 1991), and agency and assertiveness are valued traits, reflecting the independence of action and opinion (Singhal and Nagao, 1993; Kashima et al., 1995). In contrast, in predominantly collectivistic cultures, as for example seen in East Asian countries, people typically perceive themselves as interdependent (connected to others), and the self is to a much greater degree defined in terms of group memberships, relationships to family and friends as well as social roles (Markus and Kitayama, 1991; Triandis, 1993). Similarities with others and common goals are more important than in individualistic cultures (Trafimow et al., 1991). Harmony is highly valued and conflict is avoided and even suppressed (Ohbuchi et al., 1999; Carnevale and Leung, 2001; Tinsley and Brett, 2001; Cai and Fink, 2002).

These global differences in self-construal between individualistic and collectivistic cultures do not preclude further variation between individuals within these cultures (Na et al., 2010). Gender has been put forward as a prominent source of variation in self-construal, with on average a more interdependent self-construal in females (Cross and Madson, 1997). This is associated with a greater tendency to create close, intimate relationships, to take the perspective of others, and to maintain harmony and avoid conflict. Males on the other hand construct a more independent self-construal, accompanied by a preference for autonomy and emotional detachment from others. This distinction has been further refined by Baumeister and Sommer (1997), who argued that males equally construct the self as interdependent, but seek to connect to a broader group with a shared identity (e.g., supporting sports teams, patriotism, fraternities, etc.). Where females primarily seek intimate connections in a number of close personal dyadic relationships, males orient to a larger sphere by seeking to connect with a broader group; effectively seeking a larger number of relationships, but investing less in one on one interactions. The difference in interdependence between females and males has been referred to as the relational vs. collective distinction within interdependence (e.g., Kashima et al., 1995; Gabriel and Gardner, 1999; Maddux and Brewer, 2005). This has been demonstrated in a number of studies. For example, in an online trust-dilemma game, females were more trusting toward individuals with whom they shared a relationship or the potential of a future relationship, whereas men primarily trusted individuals if they were from the same social group (Maddux and Brewer, 2005). This is in line with the notion that compared to males, females tend to foster close relationships and prioritize harmony maintenance, conflict avoidance and togetherness more (Kashima et al., 1995; Cross and Madson, 1997). Similarly, Van Vugt et al. (2007) found that in males, but not in females, cooperation in a social dilemma game was increased when group cohesion was triggered by manipulating intergroup competition between the university they attended and a rival university.

The way individuals perceive themselves in relation to others influences their cognition, emotion and motivation (Markus and Kitayama, 1991; Nisbett et al., 2001; Grossmann et al., 2012). With regard to decision-making, individuals with an independent self-construal for example may rely on a cost-benefit strategy, which maximizes the benefit of one's choice for personal gain (Weber and Hsee, 2000). In contrast, individuals with an interdependent self-construal may be inclined to use role-based strategies in decision-making, focusing on the rules and expectations that come with certain social roles with the aim of fostering social connectedness (March, 1994; Weber and Hsee, 2000; Weber et al., 2005). There is indeed some empirical evidence linking independent and interdependent self-construals with different social decision-making strategies (Howard et al., 2007; Van Prooijen et al., 2008; Emonds et al., 2011). For example, in one cross-cultural study, individuals with high levels of independent self-construals displayed more competitive and less cooperative strategies than those with high levels of interdependent self-construals, independent of cultural group (European-American and Japanese) (Oetzel, 1998). Similarly, in a study investigating the relationships between self-construal, cultural background, gender, and conflict styles, Ting-Toomey et al. (2001) found that self-construal provided a better explanation for conflict style than gender and cultural background.

Experimental social dilemmas are well-suited to investigate the motives underlying social decision-making, because their design allows for quantifying the consequences of different choices for the individual and the other person(s) involved. Social dilemmas can be defined by two characteristics: (a) individuals receive higher payoffs for making selfish choices, and (b) everyone involved receives lower payoffs if everyone makes selfish choices (Weber et al., 2004). There is abundant evidence that decision-making in social dilemmas is not only influenced by individual monetary gain, but also by the moral cost or benefit associated with the action (Levitt and List, 2007; Sanfey, 2007; Rilling et al., 2008; Tabibnia et al., 2008; Balliet et al., 2009).

A widely used social dilemma is the Ultimatum Game (e.g., Roth et al., 1991; Botelho et al., 2000; Henrich et al., 2001; Gil-White, 2004; Hoffmann and Tee, 2006; Chuah et al., 2007, 2009; Güth and Kocher, 2014). This is a two-player social dilemma where a proposer suggests a division of a certain amount of money, which the responder can either accept or reject—with rejection resulting in neither party getting paid. The Ultimatum Game is a simple game: the participants make a single choice. Yet, the choice to accept or reject is a complex interplay between motivations such as inequality aversion, negative reciprocity, and costly punishment (Güth and Kocher, 2014). It is likely that these motivations will differ between people who view themselves and their actions, goals and priorities as independent from others; compared to people that construe an interdependent self-construal, viewing themselves as more connected to others, with shared goals and priorities.

Indeed, there is evidence for cultural and gender differences in the Ultimatum Game (Solnick, 2001; Oosterbeek et al., 2004; Eckel and Grossman, 2008; Espinosa and Kovářík, 2015). With regard to culture, a meta-analysis of 37 papers from 25 countries concluded that the average rejection rate of Asian responders was higher than of responders in the U.S., albeit with large heterogeneity between studies (Oosterbeek et al., 2004). However, the review was not able to establish a link between differences in rejection rate per country and cultural constructs as individualism. With regard to gender, findings have also been inconsistent. Using an Ultimatum Game design, Solnick (2001) reported that females were less likely compared to males to accept low offers, while Eckel and Grossman (2001) found that they wore more likely to accept such offers. Such inconsistencies are likely to be due to situational constraints. In a review of six social dilemma studies, Espinosa and Kovářík (2015) concluded that females compared to males displayed more prosocial behavior in experimental designs that highlight social cues. In contrast, males, but not females showed less prosocial behavior in decisions which were taken after deliberate processing compared to more spontaneous decisions (Espinosa and Kovářík, 2015). However, studies comparing either cultural or gender groups cannot address the underlying mechanisms for why differences are found (Oyserman, 2011; Oyserman et al., 2014). Rather than comparing groups that are supposed to differ in self-construal, or measuring individuals' trait self-construal and relating that to their decision-making, a more direct approach is to use priming methods to induce interdependent or independent mindsets and investigate the downstream consequences on fairness considerations in the Ultimatum Game.

Various techniques are available to prime interdependent or independent mindsets in the laboratory. For instance, Gabriel and Gardner (1999) used pronoun circling to prime an either independent or interdependent self-construal. Instructing North American participants to circle interdependent pronouns (we, ours) in a word search describing a trip to the city, resulted in a shift of world views reflecting collectivist social values and judgments, compared to when instructed to circle independent pronouns (I, mine). Trafimow et al. (1991) primed self-construal mindsets by instructing participants to think for 2 min what made them either different from their close friends and family (the independent condition) or what makes them similar (interdependent condition). Following the Similarities and Differences With Friends and Family prime, they were asked to complete 20 open-ended statements that started with “I am.” (Kuhn and McPartland, 1954). The participants who received instructions to think about what made them different from their close others reported more self-oriented responses reflecting personal attributes (e.g., I am clever, tall, etc.), while participants who received instructions to think about what made themselves similar reported more other-oriented responses reflecting social roles or qualities (e.g., I am a daughter, I am thoughtful of other's needs).

In the following, we report three studies that investigated the downstream consequences of independent and interdependent mindsets on fairness considerations, over and above individuals' trait self-construal. Participants were first primed with an independent or interdependent mindset, and then presented with a single 7-3 offer by a proposer in an Ultimatum Game. The 7-3 offer was used because prior work shows that the offer borders between being considered fair or unfair; most participants will accept a division of 50 – 40% of the total stake, while offers of less than 20% will most likely be rejected (e.g., Güth and Kocher, 2014). The 7-3 offer balances between triggering the motivation of monetary gain, and fairness considerations: while a division of 30% can be considered “unfair,” it is also better than receiving nothing. To exclude unwanted effects from proposer characteristics, we presented the offer as coming from an anonymous other. At the end of the studies participants completed a self-construal questionnaire. We predicted that an accessible interdependent mindset would result in a lower rejection rate of the Ultimatum Game offer, because it has been shown to bring connectedness and harmony to mind compared to an accessible independent mindset, that is shown to bring uniqueness and competitiveness to mind (Utz, 2004; Kim et al., 2006; Lee et al., 2010; Mourey et al., 2013). We predicted these effects to occur over and above participants' trait self-construals, but included trait self-construal in the analyses to explore possible interaction effects. In addition, we included gender for two reasons. First, an extensive collection of previous research has pointed out the role of gender in social decision-making (e.g., Eckel and Grossman, 2008; Croson and Gneezy, 2009; Espinosa and Kovářík, 2015). Second, gender has been attributed as a key factor in determining variations in self-construal (e.g., Cross and Madson, 1997).

## Study 1

In the first study we tested our predictions as detailed next by using the well-known and often used Similarities and Differences with Friends and Family task (SDFF) as the priming task (Trafimow et al., 1991). Previous research has established that this task can effectively prime self-construal (Trafimow et al., 1991; Ybarra and Trafimow, 1998; Holland et al., 2004; Oyserman and Lee, 2008; Lee and Jeyaraj, 2014). Thinking of similarities with close others decreases subjective interpersonal distance, while thinking of differences enhances such distance. It has successfully been applied in a variety of experimental setups, showing consistent moderate to high effect sizes (Oyserman and Lee, 2008). In our study participants were randomized into one of the three conditions: no-prime control, independent mindset, and interdependent mindset condition. Following the prime, they were presented with the Ultimatum Game offer: participants were asked whether they would accept or reject a 10 euro division, in which they are allocated the marginal share of 3 euro. Our predictions were threefold. First, we predicted that the participants in the interdependent condition would be more likely to accept the offer than participants in the independent condition. Second, given that the study was conducted in the Netherlands, which is classified as a typically individualistic society where people are likely to have a trait independent mindset (Hofstede et al., 1991), we expected no differences in the rejection rates between those in the no-prime control and those in the independent mindset condition. Third, we predicted that these mindset effects would be shown over and above participants' trait self-construals.

### Methods

#### Participants

A total of 226 university students participated in the study (47% male; 69% Dutch; the other 31% consisted of a diverse and fractured population: the largest subpopulations were from Suriname (4.4%), Morocco (4.4%) Turkey (4%), Iran (2.2%), and Germany (2.2%)^1^; *M*_age_ = 22.6, *SD* = 5.8, age range: 18–33 years). Participants were randomly assigned into one of the three conditions: independent-mindset condition (*n* = 75; 47% male), interdependent-mindset condition (*n* = 75; 48% male), and no-prime control condition (*n* = 76; 47% male). The study allowed advanced registration online, or walk-ins. As the experiment was conducted in Dutch, students were required to speak Dutch fluently. Active informed consent was obtained, and experimental procedures were approved by the ethical committee of the Faculty of Behavioural and Movement Sciences of the Vrije Universiteit Amsterdam (VUA). As detailed below, participants were paid for participation.

#### Procedure and materials

The study consisted of a prime followed by a one-shot Ultimatum Game and self-construal questionnaire, which were all computerized. Before participation, participants filled out a consent form, which described the study as a brief investigation of decision-making. The study started with a welcome screen and brief instructions about the Ultimatum Game. Participants were explained they would be presented with a proposal for a 10 euro split from an anonymous other student, who was randomly selected from the previous participant pool. They were also told that if they accepted the offer, a division would be made according to the proposed offer on top of the base fee; if they rejected nobody would receive an additional bonus. They were then told that they would first start with a neutral task, to clear their mind before commencing with the game.

##### Priming

The intermittent task framed as neutral, was the SDFF prime adopted from Trafimow et al. (1991). Participants in the independent-mindset condition were asked to think and write down for 2 min what made them different from their close friends and family. Participants in the interdependent-mindset condition were asked to write what they thought made them similar. In the no-prime control condition participants proceeded directly with the Ultimatum Game.

##### Ultimatum game

The social dilemma was a one-shot Ultimatum Game. The dependent variable was rejection rate: participants had to decide whether to accept or reject an offer where the anonymous proposer would receive 7 euro, and the participant 3.

##### Self-construal scale

The Ultimatum Game was followed by a trait self-construal scale (TSC) consisting of 10 items, of which half measured trait independent self-construal (e.g., “I don't care what other people think about me, as long as I am happy”; α = 0.71) and half measured trait interdependent self-construal (e.g., “Relationships with others are more important than my own accomplishments”; α = 0.75). The scale consisted of a modified version of the Oyserman (1993) scale, translated to Dutch. Participants rated themselves on a 7-point scale (1 = *does not describe me at all* to 7 = *describes me very well*).

Finally, participants were asked to indicate their age, gender, study enrollment, and ethnicity for demographic analysis. Participants were then debriefed; they were informed that the offer was fixed, and the neutral task consisted of a prime designed to make an independent or interdependent self-construal salient in the different priming conditions. At the end of the study participants were thanked and paid. Payment consisted of a base fee of €2, with a bonus of €0.30 if they accepted the proposed offer.

#### Analyses plan

A logistic regression analysis was conducted to analyze whether a model with the predictors condition (independent prime, interdependent prime and no-prime control) and gender provided a better fit at predicting the binary outcome of rejection rate (accept or reject) on the Ultimatum Game than a null (intercept-only) model. A logistic regression model with categorical predictors examines pairwise interactions of rejection rate of gender across the different conditions; only comparisons between cells are analyzed (e.g., the difference between priming interdependence vs. independence for males, or the difference between males and females in the interdependent-prime condition), and does not compute the overall effect of condition or gender as an ANOVA analysis.

Pairwise interactions were examined to analyze rejection rate of gender across the different conditions. The odds ratio are reported with 95% confidence intervals. The estimated probabilities of rejection rate were indexed by the partial logistic regression coefficients (*B*-values) of the means of gender within the different conditions, means of conditions within gender, and the interaction between gender and condition.

In order to examine the extent to which trait levels of independence and interdependence moderated the outcome of condition on the Ultimatum Game, a ratio score of independence and interdependence scores was computed and included as a predictor in the analysis. A higher score on TSC indicated a higher ratio of interdependence. All two- and three-way interaction effects were included in the model.

### Results

#### Descriptives

Overall, 42.7% of the participants in the interdependent-mindset condition rejected the offer (RO) in the Ultimatum Game, compared to 58.7% of the participants in the independent-mindset condition, and 56.6% of the participants in the control condition. To examine whether participants differed in trait levels of independence or interdependence as a function of condition and gender, a two-way ANOVA was performed with TSC as the dependent variable. Only a main effect of gender was found, *F*_(1, 220)_ = 8.39, *p* = 0.004, Cohen's *d* = 0.39. Overall, males reported a higher ratio of independence to interdependence (*M* = −0.23, *SD* = 0.69) than females (*M* = –0.02, *SD* = 0.38). TSC did not differ between the three conditions.

#### The ultimatum game

We conducted a logistic regression including condition, gender, TSC, and all two- and three-way interaction effects as predictors (Table 1). The analysis revealed a non-significant three-way interaction between the independent and interdependent-mindset conditions with gender and TSC: Wald $\chi_{\left(1,\;\text{N}\;=\;226\right)}^{2}$ = 2.92, *p* = 0.09. The two-way interaction between gender and the independent-mindset and interdependent-mindset conditions was significant [Wald $\chi_{\left(1,\;\text{N}\;=\;226\right)}^{2}$ = 5.17, *p* = 0.02].

**Table 1:** **Study 1: Regression model for the predictors condition, gender, CO; and the two- and three-way interaction effects on rejection rate**.

	**β**	***SE***	**Wald**	***p***	***e*^β^**	**95% CI**
**FEMALES**						
Condition^a^ (IND)	1.31	0.48	7.31	0.007^**^	3.69	[1.43, 9.49]
Condition^a^ (CTRL)	0.97	0.47	4.23	0.04^*^	2.63	[1.05, 6.60]
TSC (IND)	0.94	0.84	1.26	0.26	2.57	[0.49, 13.32]
TSC (INTER)	−0.32	1.06	0.09	0.76	0.73	[0.09, 5.85]
TSC (CTRL)	1.30	1.49	0.76	0.38	3.66	[0.20, 67.96]
TSC × Condition^a^ (IND)	1.26	1.36	0.87	0.35	3.54	[0.25, 50.40]
TSC × Condition^a^ (CTRL)	1.62	1.83	0.78	0.38	5.04	[0.14, 182.54]
**MALES**						
Condition^a^ (IND)	−0.32	0.53	0.37	0.54	0.72	[0.26, 2.04]
Condition^a^ (CTRL)	−0.13	0.54	0.06	0.81	0.88	[0.31, 2.52]
TSC (IND)	−0.78	0.74	1.12	0.29	0.46	[0.11, 1.94]
TSC (INTER)	1.80	1.63	1.21	0.27	6.02	[0.25, 147.52]
TSC (CTRL)	−0.21	0.78	0.08	0.78	0.81	[0.17, 3.75]
TSC × Condition^a^ (IND)	−2.58	1.79	2.07	0.15	0.08	[0.01, 2.54]
TSC × Condition^a^ (CTRL)	−2.01	1.81	1.23	0.27	0.13	[0.01, 4.65]
**INDEPENDENT CONDITION**						
Gender^b^	−0.64	0.50	1.59	0.21	0.53	[0.20, 1.42]
Gender^b^ × TSC	−1.72	1.12	2.38	0.12	0.18	[0.02, 1.60]
**INTERDEPENDENT CONDITION**						
Gender^b^	0.99	0.51	3.81	0.05^*^	2.70	[1.00, 7.32]
Gender^b^ × TSC	2.12	1.95	1.18	0.28	8.30	[0.18, 377.59]
**CONTROL CONDITION**						
Gender^b^	−0.10	0.50	0.04	0.84	0.90	[0.34, 2.41]
Gender^b^ x TSC	−1.51	1.68	0.81	0.37	0.22	[0.01, 5.97]
**GENDER BY CONDITION INTERACTIONS**						
Gender^b^ × Condition^a^ (IND)	−1.63	0.72	5.17	0.03^*^	0.20	[0.05, 0.80]
Gender^b^ × Condition^a^ (CTRL)	−1.10	0.71	2.37	0.12	0.33	[0.08, 1.35]
Gender^b^ × TSC × Condition^a^ (IND)	−3.84	2.25	2.92	0.09^+^	0.02	[0, 1.76]
Gender^b^ × TSC × Condition^a^ (CTRL)	−3.63	2.58	1.99	0.16	0.03	[0, 4.13]
Constant^a^^b^	−0.69	0.34	4.11	0.04^*^	0.50	

a *Reference category = interdependent primed*.

b *Reference category = female. IND, independent priming condition; INTER, interdependent priming condition; CTRL, control condition*.

+ *p < 0.1*,

* *p < 0.05*,

** *p < 0.01*.

**Figure 1:**
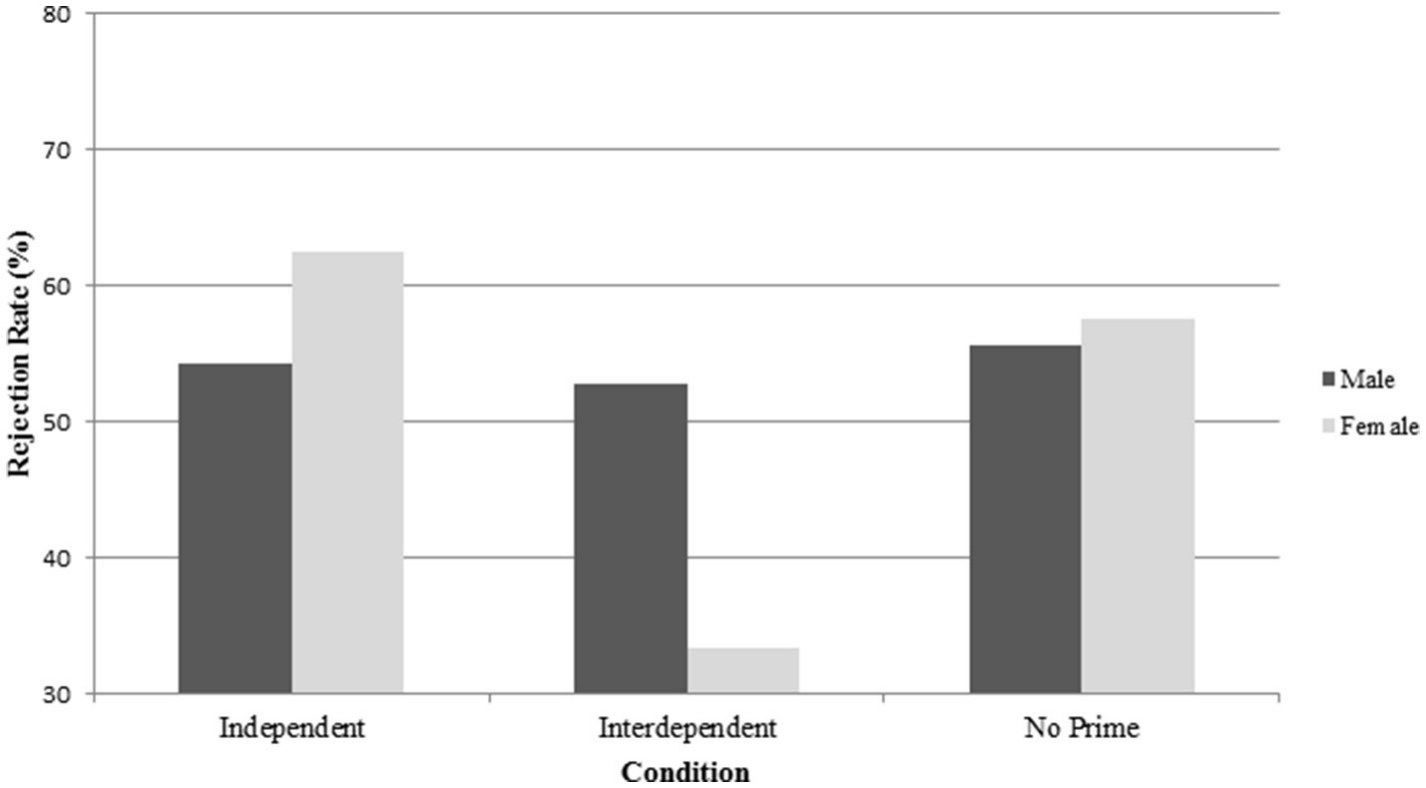
**Study 1: Average rejection rate of males and females in the priming conditions**.

The results showed that females rejected less when they were primed with interdependence (RO = 33.3%) compared to when they were primed with independence (RO = 62.5%), Wald $\chi_{\left(1,\;\text{N}\;=\;226\right)}^{2}$ = 7.31, *p* = 0.007, or when they were not primed at all (RO = 57.5%), Wald $\chi_{\left(1,\;\text{N}\;=\;226\right)}^{2}$ = 4.23, *p* = 0.04. The standardized regression values indicated that females in the interdependent condition were 3.7 times less likely to reject the offer than those in the independent condition, and 2.6 times less likely to reject than those in the control condition. Females in the independent condition did not differ from those in the control condition. Males on the other hand did not respond differently based on the different priming conditions (RO_independent_ = 54.3%, RO_interdependent_ = 52.8%, RO_control_ = 57.5%) (Figure 1).

The difference between males and females was only significant in the interdependent mindset condition, where males rejected more often than females, Wald $\chi_{\left(1,\;\text{N}\;=\;226\right)}^{2}$ = 3.81, *p* = 0.05. As expected, the interactions with trait self-construal were not significant.

### Discussion

We predicted that participants in the interdependent mindset condition would reject the offer less than those in the independent mindset and the no-prime control condition, over and above participants' trait self-construals. Our results partially support these two predictions. While an accessible interdependent mindset indeed resulted in a lower rejection rate, this was only true for females. Male participants' rejection rate of the offer did not differ as a function of condition. These findings were not influenced by participants' trait self-construals. Moreover, despite that females reported higher trait levels of interdependent self-construal, there were no differences between females and males in the no-prime control condition. These findings suggests that participants' gender affects sensitivity to the mindset prime, rather than a priori resulting in different decisions in the Ultimatum Game.

These findings are in line with a recent review concluding that there are no differences between males and females in social dilemmas with a neutral experimental framing, while cues highlighting the social context of the dilemma lead to more prosocial behavior in females (Espinosa and Kovářík, 2015). Other results also coincide with the notion that females are generally more sensitive to the relational aspects of interdependence than males (Cross and Madson, 1997; Maddux and Brewer, 2005). In our study, thinking and writing about similarities with close others could have triggered these relational aspects of interdependence, and may therefore have had a stronger effect in females than males.

In contrast, it has been suggested that males are more sensitive to the collective aspect of interdependence (Baumeister and Sommer, 1997; Gabriel and Gardner, 1999). This collectivity refers to feeling connected to peers with a shared identity or group membership. The distinction between relational and collective interdependence has been put forward to explain gender differences in sociality in terms of the male and female motivations to connect to different spheres (Baumeister and Sommer, 1997). This difference may also affect outcomes in social dilemma's. In the following two studies we further examined if the nature of the prime may have triggered the gender difference in the effect on participants fairness considerations. As Study 1 suggested that females are more than males influenced by having relational interdependence on their mind, we tested whether males would be more influenced by a prime triggering collective interdependence in Study 2.

## Study 2

We developed a new prime using a modified university mission statement. Participants read a text about collective norms of how students at the university are expected to behave among their peers. Participants were randomly assigned into one of the two conditions: independent mindset and interdependent mindset condition. Participants in the independent mindset condition read about the importance of being independent, having one's own opinion, being unique, and having personal goals. Participants in the interdependent mindset condition read about the importance of being honest, equal, social, and interpersonal. The crucial difference between this new prime and the previous Similarities and Differences prime is that in Study 2 we tap into a collective identity (that of the university to which the students belong). Following the prime, participants were again asked whether they would accept or reject a 7-3 offer in an Ultimatum Game.

Our predictions were twofold. First, we predicted that the participants in the interdependent condition would be more likely to accept the offer than participants in the independent condition. Yet, given that the interdependent prime now elicits collective rather than relational interdependence, we expected that the condition effect would be more pronounced in males than females. Second, we predicted that these mindset and gender effects would be shown over and above participants' trait self-construals. As we had established a baseline rejection rate in a no-prime control condition, we did not include a control condition in order to allocate the available population to the two priming conditions to ensure enough statistical power.

### Methods

#### Participants

A total of 180 students participated in the study (39% male; 81% Dutch, *M*_age_ = 22.50, *SD* = 2.77, age range: 18–29 years). Both the independent- and interdependent-mindset conditions consisted of 90 participants (35 males, 55 females). Due to characteristics of the prime, participants were required to be a registered student, as well as speak Dutch fluently. Active informed consent was obtained, and experimental procedures were approved by the ethical committee of the Faculty of Behavioural and Movement Sciences. Participants were not paid, but received a candy bar upon completion.

#### Materials and procedure

The study was conducted in the seating area of the main building of the Vrije Universiteit. The location provided access to a large and varied student population. The experiment consisted of a printed booklet, that could be completed within a few minutes. The booklets of both conditions were randomized and conducted in a 2-week period. Prior to the last day of data collection, the collected data were summarized over gender and condition in order to balance the remaining booklets.

##### Priming

A new prime was devised, consisting of a modified version of the VUA mission statement. Instead of the projected long term goals and values of the university, the statement was modified to represent values consistent with an independent or interdependent self-construal. In addition, the VUA logo was included, with a modified slogan (the standard slogan is “looking forward”). In the independent-prime condition the text now read (translation from Dutch):“*The VU has a clear policy concerning the goals and values on campus. The VU emphasizes that students should develop* independence *and having their* own opinion. *VU students are encouraged to develop skills that make them a* unique *individual. Students should maintain* personal goals.” In the interdependent-prime condition, the underlined words were replaced by: honesty, equality, social, and interpersonal (underlining for clarification, not presented during experimental presentation). The VU logo was positioned in the top right corner, with the underlying text “*everyone unique”* in the independent-mindset condition, and “*everyone equal”* in the interdependent-mindset condition. The statement was followed by the question “*Do you agree with the following statements:” “Do you recognize the VU students in the description above?”* and “*Do you recognize yourself in the description above?”* Answers were given on a 5-point Likert scale (1 = “*not at all”* to 5 = “*completely”)*^2^.

##### Ultimatum game

The social dilemma was again a one-shot Ultimatum Game offer with rejection rate as binary dependent variable. Participants had to decide whether to accept or reject an offer of 7 for the anonymous proposer, and 3 for the participant.

##### Self-construal scale

The Ultimatum Game was followed by the same TSC as in Study 1 consisting of 10 items; 5 independent items (α = 0.69) and 5 interdependent items (α = 0.63).

##### Demographics

Finally, participants were asked to complete their age, gender and ethnicity, as well as provided the option of leaving a remark concerning the study. Participants were debriefed upon completion and awarded a candy bar for their participation.

#### Analyses plan

As in Study 1, we conducted a regression analysis with condition, gender and TSC as predictors, and rejection rate as binary outcome to examine whether this model provided a better fit than an intercept-only model. All two- and three-way interactions were included.

### Results

#### Descriptives

Overall, 61% of the participants in the interdependent-mindset condition rejected the offer (RO) in the Ultimatum Game, compared to 57% of the participants in the independent-mindset condition. As in Study 1, a two-way ANOVA was performed to examine whether trait levels of independence and interdependence differed as a function of gender and condition. Females reported a higher ratio of interdependence to independence (*M* = 0.05, *SD* = 0.21) than males (*M* = –0.05, *SD* = 0.25), *F*_(1, 176)_ = 7.37, *p* = 0.007, Cohen's *d* = 0.41. TSC did not differ between the conditions.

#### Analysis of the ultimatum game

**Figure 2:**
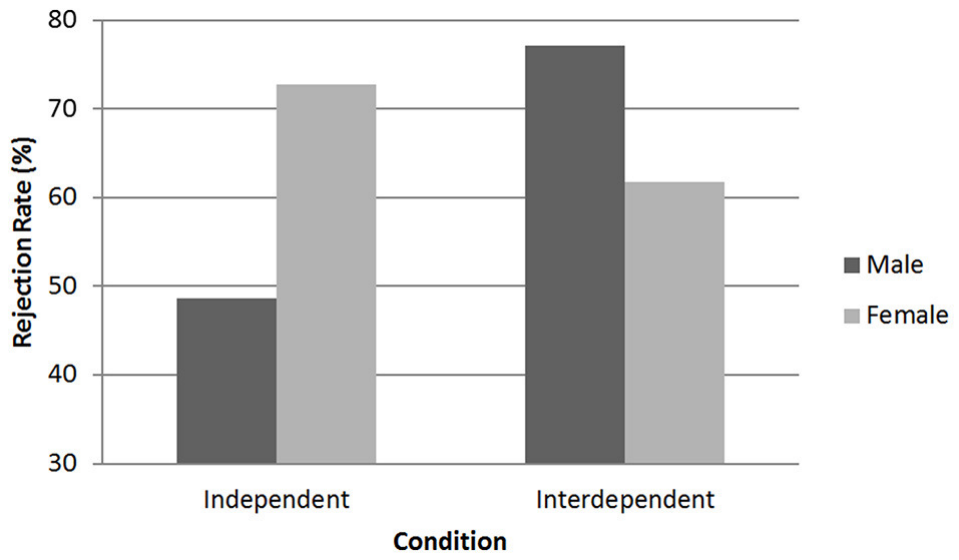
**Study 2: Average rejection rate of males and females in the priming conditions**.

A logistic regression with the predictors gender, condition, TSC including all two- and three-way interactions was conducted (Table 2). As in Study 1, the analysis revealed a significant interaction between gender and condition: Wald $\chi_{\left(1,\;\text{N}\;=\;180\right)}^{2}$ = 5.69, *p* = 0.02. The interaction effect indicated males and females displayed a different pattern of fairness consideration based on the priming conditions. Specifically, males rejected the offer more often when primed with interdependence (RO = 77.1%) compared to independence (RO = 48.6%), Wald $\chi_{\left(1,\;\text{N}\;=180\right)}^{2}$ = 5.39, *p* = 0.02. Rejection rate of females on the other hand was not significantly influenced by condition (*p* = 0.38); females in the independent condition rejected the offer more often when primed with independence (RO = 72.7%) compared to interdependence (RO = 61.8%) (Figure 2). When primed with independence, females were three times more likely to reject the offer than males, Wald $\chi_{\left(1,\;\text{N}\;=180\right)}^{2}$ = 5.63, *p* = 0.02. Rejection rate between males and females did not differ in the interdependent mindset condition. Similar to Study 1, trait self-construal did not influence the effects.

**Table 2:** **Study 2: Regression model for the predictors condition, gender, CO, and the two- and three-way interaction effects on rejection rate**.

	**β**	***SE***	**Wald**	***p***	***e*^β^**	**95% CI**
**FEMALES**						
Condition^a^	0.37	0.43	0.78	0.38	1.46	[0.63, 3.35]
TSC (IND)	0.13	0.73	0.03	0.86	1.14	[0.27, 4.78]
TSC (INTER)	−1.04	0.83	1.57	0.21	0.35	[0.07, 1.80]
TSC × Condition^a^	1.18	1.11	1.13	0.29	3.24	[0.37, 28.38]
**MALES**						
Condition^a^	−1.31	0.56	5.39	0.02^*^	0.27	[0.09, 0.82]
TSC (IND)	−1.47	1.93	0.58	0.45	0.23	[0.01, 10.13]
TSC (INTER)	−2.99	1.95	2.36	0.12	0.05	[0.01, 2.29]
TSC × Condition^a^	1.53	2.74	0.31	0.58	4.60	[0.02, 993.83]
**INDEPENDENT CONDITION**						
Gender^b^	−1.15	0.49	5.63	0.02	0.32	[0.12, 0.82]
Gender^b^ × TSC	−1.60	2.06	0.60	0.44	0.20	[0.01, 11.53]
**INTERDEPENDENT CONDITION**						
Gender^b^	0.53	0.51	1.08	0.30	1.70	[0.62, 4.66]
Gender^b^ × TSC	−1.95	2.12	0.85	0.36	0.14	[0.01, 9.06]
**GENDER BY CONDITION INTERACTIONS**						
Gender^b^ × Condition^a^	−1.69	0.71	5.69	0.02^*^	0.19	[0.05, 0.74]
Gender^b^ × TSC × Condition^a^	0.35	2.96	0.01	0.91	1.42	[0.01, 468.01]
Constant^a^^b^	0.60	0.30	4.06	0.04^*^	1.82	

a *Reference category = interdependent primed*.

b *Reference category = female. IND, independent priming condition; INTER, interdependent priming condition*.

* *p < 0.05*.

### Discussion

Study 2 further explored the influence of gender on fairness considerations as a function of one's self-construal mindset. Where Study 1 showed that females rejected the Ultimatum Game offer less when primed with relational interdependence than independence, Study 2 examined whether males would be influenced by the mindset conditions when collective, rather than relational interdependence was activated. Indeed, in line with our predictions we found that differences in fairness considerations as a function of interdependent and independent mindsets were more pronounced in males in Study 2 than in Study 1. However, the direction of the effect in males was opposite to our expectation: activating a collective interdependent compared to an independent mindset *increased* rejection rate in males.

A possible *post-hoc* explanation for this unexpected effect may be that the collective interdependent prime elicited norm enforcing behavior in males. Highlighting a collective identity has been associated with a greater tendency for norm enforcement (Horne, 2007). Likely, receiving an offer that can be considered unfair violates with the prescribed collective norm of acting social and keeping others' goals and priorities in mind, as phrased in the interdependent prime. Baumeister and Sommer (1997) have argued that compared to females, males have a greater sensitivity toward the collective aspects of interdependence. Following a different sensitivity to the mindset prime, receiving an unfair offer may have triggered norm enforcing behavior in males, resulting in a higher rejection rate.

Taken together, Study 2 suggests that males and females differ in their sensitivity to a collective mindset prime. In Study 3 we tested the robustness of this effect by replicating the study in a controlled laboratory environment, at two different universities. Additionally, we added a manipulation check to examine whether differences between the conditions could be contributed to the activation of different self-construal mindsets.

## Study 3

Study 3 was nearly identical to Study 2. Differences were that the study took place in the laboratory and at two universities and a manipulation check was added. Participants were randomly assigned into the independent or interdependent mindset condition. They started by reading the modified university mission statement, after which they were asked whether they would accept or reject a 7-3 offer in an UG, and to complete “I am” statements before providing demographics. We expected that the findings of Study 2 would be replicated: males would reject relatively more when primed with interdependence, while females would reject more when primed with independence.

### Methods

#### Participants

The data were collected at two locations: the University of Amsterdam (UvA) (*N* = 83, 29% male), and the Vrije Universiteit Amsterdam (VUA) (*N* = 97, 47% male). In total 180 students participated in the study (39% male, 86% Dutch, *M*_age_ = 21.44, *SD* = 2.75, age range: 18–31 years). Both the independent- and interdependent-mindset conditions consisted of 90 participants (35 males, 55 females). Due to characteristics of the prime, participants were required to be a registered student, as well as speak Dutch fluently. Active informed consent was obtained, and experimental procedures were approved by the ethical committee of the Faculty of Behavioural and Movement Sciences. Participants were paid identical to Study 1.

#### Materials and procedure

The study was conducted in two locations. The first location was in the lab of the VUA, and the second in the lab of the UvA. The experiment was constructed with Qualtrics software (version 26,568). Participants were randomized into the two priming conditions, taking gender into account to create even cells. Similar to Studies 1 and 2, the experiment consisted of the prime followed by a one-shot, two-way Ultimatum Game and self-construal questionnaire. Before participation, participants filled out a consent form, and read an information sheet containing instructions about the Ultimatum Game. They were then told that they would first start with a neutral task, to clear their mind before commencing with the game. However, two modifications were included based on the novelty of the Mission prime. First, after the Ultimatum Game offer was presented, participants were asked to recall the content of the mission prime presented earlier. The question served as a check to establish whether the participants had properly read the prime, as well as allowing us to infer what they had summarized as the core meaning of the text. This was then followed by the second modification, which consisted of the manipulation check. The study was completed by the TSC and demographics as described in Studies 1 and 2.

##### Priming

The prime was the same Mission prime employed in Study 2, consisting of an independent and interdependent condition. In the independent condition students were told the university encourages independence and having an own opinion, as well as being unique and focusing on personal goals. In the interdependent mindset condition students were told the university values honesty, equality, and that they were expected to act social and keep others' goals in mind. Following the prime, participants were asked to rate on a 5-point scale to which degree the content applied to themselves and their peers.

##### Ultimatum game

The social dilemma was again a one-shot Ultimatum Game offer of with rejection rate as binary dependent variable. Participants decided whether to accept or reject an offer of 7 for the anonymous proposer, and 3 for the participant.

##### Manipulation check

A shorter version of Kuhn and McPartland's (1954) Twenty Statements Test was included as a manipulation check for the self-construal prime. Participants were asked to complete 10 (instead of twenty) open-ended sentences starting with “I am.” Their responses were coded as either independent or interdependent as follows: responses that described a personal trait (e.g., I am intelligent, pretty, tall) or feeling (I am happy, stressed) were encoded as independent, while responses that described a social role (e.g., I am a partner, sister), group membership (e.g., I am a psychology student, male, or inhabitant of Amsterdam) or social qualities (e.g., I am empathetic, I am someone who cares about others) were coded as interdependent (Agrawal and Maheswaran, 2005; Lee and Jeyaraj, 2014).

##### Self-construal scale

The Ultimatum Game was followed by the same self-construal scale (TSC) as in Study 1 consisting of 10 items; 5 independent items (α = 0.63) and interdependent items (α = 0.60).

##### Demographics

Finally, participants were asked to complete their age, gender and ethnicity, as well as provided the option of leaving a remark concerning the study. Participants were debriefed upon completion.

#### Analyses plan

As in Studies 1 and 2, we conducted a regression analysis with condition, gender and TSC as predictors to examine whether this model provided a better fit than an intercept-only model. All two- and three-way interactions were included. Initially we conducted a regression analysis to examine whether the outcomes differed as a function of location (VUA lab vs. UvA lab. There were no significant differences in overall rejection rate based on location, nor was there an interaction effect between condition and location, or between gender, condition and location. Therefore, the similar direction of effects provided us with enough confidence to merge the data sets from the different locations.

Responses in the manipulation check (Twenty Statements Task) were analyzed by conducting a two-way ANOVA with gender and condition as factors. The number of interdependent^3^ responses was divided by the total number of responses from each participant (Trafimow et al., 1991; Lee and Jeyaraj, 2014).

### Results

#### Descriptives

As in Study 1, a two-way ANOVA was performed to examine whether trait levels of independence and interdependence differed as a function of gender and condition. Only a main effect of gender was established: *F*_(1, 176)_ = 9.441, *p* = 0.002, Cohen's *d* = 0.46. Females reported a higher ratio of interdependence (*M* = 0.09, *SD* = 0.40) compared to males (*M* = –0.08, *SD* = 0.21). There was no significant difference between the priming conditions.

#### Analysis of the manipulation check

A two-way ANOVA with gender and condition as factors, revealed that participants in the interdependent-mindset condition reported a higher number of interdependent-relevant responses (*M* = 1.35, *SD* = 0.17) than participants in the independent-mindset condition (*M* = 1.27, *SD* = 0.17), *F*_(1, 168)_ = 10.84, *p* = 0.001, *d* = 0.51. Additionally, females reported more interdependent-relevant responses (*M* = 1.34, *SD* = 0.17) than males (*M* = 1.26, *SD* = 0.17), *F*_(1, 168)_ = 12.14, *p* = 0.001, Cohen's *d* = 0.54.

#### Analysis of the ultimatum game

A binary logistic regression was conducted with condition, gender, TSC, and all two- and three-way interactions. The analysis revealed a three-way interaction between gender, TSC and condition: Wald $\chi_{\left(1,\;\text{N}\;=\;180\right)}^{2}$ = 6.08, *p* = 0.01. The three-way interaction indicated participants' self-construal was also instrumental in determining the outcome of the decision. Based on the three-way interaction, the analysis of the hypothesized two-way interaction between gender and condition was followed up by an analysis of the interaction between self-construal and condition for males and females separately, and between gender and self-construal for the two conditions separately.

**Figure 3:**
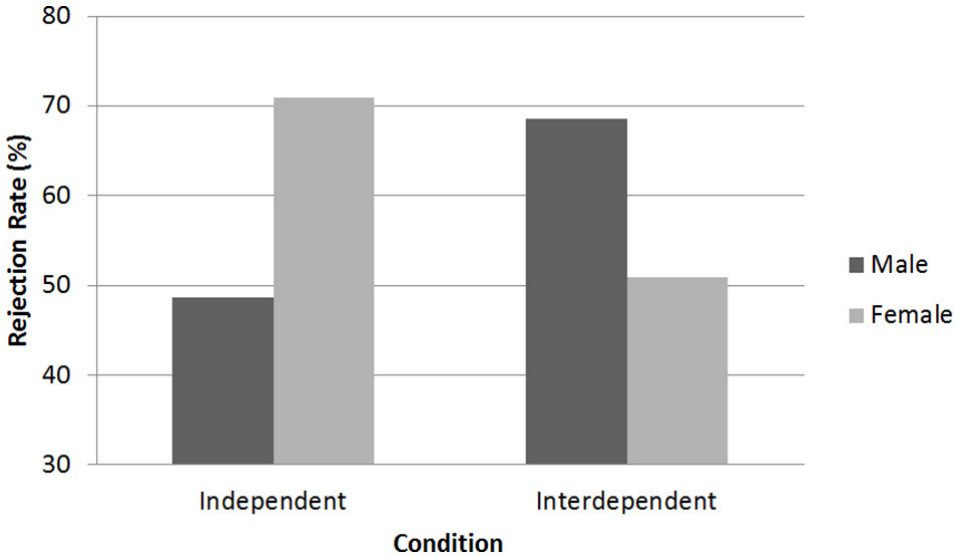
**Study 3: Average rejection rate of males and females in the priming conditions**.

Similar to Studies 1 and 2, we established a significant interaction effect between gender and the priming conditions, Wald $\chi_{\left(1,\;\text{N}\;=\;180\right)}^{2}$ = 4.10, *p* = 0.04 (Table 3), indicating males and females displayed a different pattern over the two priming conditions. Males rejected the offer more often when primed with interdependence (RO = 68.6%) compared to independence (RO = 48.6%). While the effect size was similar to Study 2, the effect was non-significant (*p* = 0.38). Females on the other hand rejected the offer more often when primed with independence (RO = 70.9%) compared to interdependence (50.9%) (Figure 3). Wald $\chi_{\left(1,\;\text{N}\;=\;180\right)}^{2}$ = 4.66, *p* = 0.03; females primed with interdependence were 2.5 times more likely to accept the offer, compared to when primed with independence. When comparing between gender, the difference between males and females in the independent-mindset condition was significant, Wald $\chi_{\left(1,\;\text{N}\;=\;180\right)}^{2}$ = 4.42, *p* = 0.04; when primed with independence, females were 2.6 times more likely to reject the offer than males. The difference between gender in the interdependent condition was not significant.

**Table 3:** **Study 3: Regression model for the predictors condition, gender, CO; and the two- and three-way interaction effects on rejection rate**.

	**β**	***SE***	**Wald**	***p***	***e*^β^**	**95% CI**
**FEMALES**						
Condition^a^	0.91	0.42	4.66	0.03^*^	2.47	[1.09, 5.62]
TSC (IND)						
TSC (INTER)	0.40	1.36	0.09	0.77	1.49	[0.10, 21.61]
TSC × Condition^a^	−1.25	1.95	0.41	0.52	0.29	[0.01, 13.25]
**MALES**						
Condition^a^	−0.47	0.54	0.77	0.38	0.63	[0.22, 1.78]
TSC (IND)						
TSC (INTER)	−7.75	3.45	5.04	0.03^*^	0.01	[0.01, 0.37]
TSC × Condition^a^	8.95	3.64	6.03	0.01^**^	7672.63	[6.09, 9672783.75]
**INDEPENDENT CONDITION**						
Gender^b^	−0.96	0.46	4.42	0.04^*^	0.38	[0.16, 0.94]
Gender^b^ × TSC	2.04	1.82	1.26	0.26	7.70	[0.22, 271.83]
**INTERDEPENDENT CONDITION**						
Gender^b^	0.42	0.50	0.68	0.41	1.51	[0.57, 4.06]
Gender^b^ × TSC	−8.15	3.71	4.82	0.03^*^	0.01	[0.01, 0.42]
**GENDER BY CONDITION INTERACTIONS**						
Gender^b^ × Condition^a^	−1.38	0.68	4.10	0.04^*^	0.25	[0.07, 0.96]
Gender^b^ × TSC × Condition^a^	10.19	4.13	6.08	0.01^**^	266687.42	[8.08, 880981.38]
Constant^a^^b^	0.01	0.29	0.01	0.99	1.00	

a *Reference category = interdependent primed*.

b *Reference category = female. IND, independent priming condition; INTER, interdependent priming condition*.

* *p < 0.05*,

** *p < 0.01*.

However, the gender differences were further qualified by significant two-way interactions with trait self-construal. First, there was a significant TSC × Condition interaction in males, Wald $\chi_{\left(1,\;\text{N}\;=\;180\right)}^{2}$ = 6.03, *p* = 0.01. Specifically, trait self-construal affected male decision-making in the interdependent condition, Wald $\chi_{\left(1,\;\text{N}\;=\;180\right)}^{2}$ = 5.04, *p* = 0.03, but not in the independent condition. In addition, there was a significant interaction between gender and self-construal in the interdependent mindset condition, Wald $\chi_{\left(1,\;\text{N}\;=\;180\right)}^{2}$ = 4.82, *p* = 0.03, but not in the independent condition. The two-way interactions can be explained as follows: while males rejected the offer more often when primed with interdependence, trait self-construal affected the outcome of their decision; specifically, when primed with interdependence, males with a relatively high level of independence rejected the offer more often than those with a high level of interdependence. For females however, trait self-construal did not affect the outcome in both the interdependent and independent conditions, nor did it interact with the priming conditions. Females were primarily affected by the mindset prime, and rejected more often when primed with independence compared to interdependence.

### Discussion

Our second study replicated our initial findings of the Mission prime with a one-shot Ultimatum Game. Males and females displayed an opposite pattern in the different priming conditions: males rejected the offer more often when primed with interdependence, while females rejected the offer more when primed with independence. The effect proved robust despite varying experimental conditions: a paper-and pencil hypothetical study on campus vs. a lab study with performance-contingent payment. In addition, the data from the two university labs, using adapted logo and slogan, was similar.

This time, we established a three-way interaction between trait self-construal, the priming conditions, and gender. Interestingly, trait self-construal only affected male decision-making. Males with a higher level of independence rejected the offer more when primed with interdependence compared to males with a higher level of interdependence. The interdependent-mindset prime describes that the participants (and their peers) are expected to behave in a social manner, taking each other's goals and priorities into account. Theoretically, the prime can influence fairness considerations in two ways: (1) everybody should behave socially, therefore the anonymous proposer should have proposed a more fair offer and I reject, or (2) everybody should behave socially, therefore I should accept the offer instead of punishing. Possibly, males with a higher level of independence rejected the offer more often due to the first implicit reasoning, while males with a higher ratio of interdependence inferred the second. However, an effect of trait self-construal was only observed in Study 3, and therefore this finding should be interpreted with caution.

## General discussion

We conducted three studies using self-construal primes to investigate the downstream consequences of independent vs. interdependent mindsets on fairness considerations in the Ultimatum Game. We initially hypothesized that activating an interdependent mindset would be associated with lower rejection rates compared to an independent mindset, because interdependence is connected to motivations relating to conflict avoidance, harmony maintenance and social connectedness (Markus and Kitayama, 1991; Trafimow et al., 1991; Triandis, 1993; Cross and Madson, 1997). However, the results showed that both the specific self-construal prime as well as participants' gender mattered.

In line with our hypotheses, Study 1 showed that those in the interdependent mindset condition (thinking of similarities with close others) lowered the average rejection rate compared to those in the independent mindset condition (thinking about differences with others) and the no-prime control condition—but only in females. Male decision-making behavior was not affected by the primes. In contrast, the mission primes in Studies 2 and 3 yielded a reversed effect in males: those in the interdependent mindset condition (prescribing social behavior among peers), *increased* the average rejection rate of the offer compared to those in the independent mindset condition (prescribing self-development and prioritization of own goals). In females, the independent mindset condition increased the rejection rate, compared to the interdependent condition.

Why did we find these gender differences, even though participants were cued with the same stimuli? Our findings suggest that males and females are sensitive to different aspects of a self-construal prime. Self-construal affects the motives underlying social-decision making by turning the focus on the self (independent mindset) or on the relationship with the other player (interdependent mindset) (Gardner et al., 1999). Consider the SDFF prime as used in Study 1. Here, the interdependent mindset prime highlights the *relationship* with the other player. Prior work suggests that women are more sensitive to the relational aspects of interdependence than men (Cross and Madson, 1997; Maddux and Brewer, 2005). In our Study 1, bringing close dyadic relationships to mind led women to inhibit the tendency to punish.

Now consider the mission prime as used in Study 2. Here the interdependent mindset prime highlights a *collective* identity, and prescribes a social norm (i.e., students are expected to act in a pro-social manner). Previous studies suggest that compared to women, men are more sensitive to the collective aspects of interdependence (Kashima et al., 1995; Gabriel and Gardner, 1999). In our Studies 2 and 3, the mission prime may have elicited an interpretation that *everybody* should act pro-socially, according to a collective norm. For females, this resulted in a similar pattern as Study 1—rejecting the offer relatively less when primed with interdependence compared to independence. Males on the other hand rejected the offer more often when presented with the interdependent prime. One plausible explanation is that when a collective relationship is salient, males are more likely than females to perceive unfair offers as transgressions of group norms, resulting in higher rejection rates. Prior work indeed indicates that males, more than females, are sensitive to the collective aspect of interdependence. For example, Van Vugt et al. (2007) varied intergroup competition in a public goods game, by either mentioning that performance (i.e., contributions to the public good) would be assessed at the individual level, or would be compared to other groups of rival universities. While female cooperation was equally high across the two conditions, male cooperation equaled that of females only when their collective identity was triggered. Maddux and Brewer (2005) measured levels of trust toward an anonymous other, framed as either a member of the same university, a different university, or the university where one of their friends attended (which could therefore potentially become a future relationship). Men displayed higher levels of trust to members of their own university, while women displayed more trust to the potential relationship target as well as members of their own university.

Studies 1 and 2 both demonstrated the effects of self-construal priming over and above participants' trait self-construal levels. Only in Study 3 participants' trait self-construal moderated the interaction between gender and self-construal priming. Primed with interdependence, males with a higher ratio of independence rejected the offer on average more often, whereas males with a higher ratio of interdependence rejected the offer relatively less often. Possibly, males with a higher level of independence, as assessed by the TSC, were more inclined to interpret the interdependent prime as a norm that should be enforced on others, compared to males with a relatively high level of interdependence. However, this is a *post-hoc* interpretation of a result that was not hypothesized and was observed in one study only.

### Implications and future directions

Our findings suggest that men and women differ in their sensitivity to social cues and that these differences spill over to their social decisions. As such, they contribute to the literature in several ways. First, our findings indicate the need to consider gender as a factor in priming studies. In prior studies, it is often unclear whether gender differences were taken into account or just not found. Possibly, the moderating effect of gender observed in the current studies can be ascribed to the nature of the dependent variable. Specifically, the Ultimatum Game elicits strong emotional reactions of anger and disgust (Sanfey et al., 2003; Sanfey, 2007; Rilling et al., 2008; Tabibnia et al., 2008). Future studies can usefully investigate if the gender effect occurs specifically in the context of affective social decision-making. Second, our findings shed some light on inconsistencies in the results of previous studies examining gender differences in social decision-making, which have been identified by two reviews (Eckel and Grossman, 2008; Croson and Gneezy, 2009). Eckel and Grossman (2008) argue that inconsistencies occur because the inclusion of risk in social dilemma paradigms has a more pronounced effect of decisions in women than in men, based on the notion that women may be more risk-averse than men (Byrnes et al., 1999; Charness and Gneezy, 2012). However, in a study specifically designed to test this hypothesis, higher risk-aversion in women did not result in lower rejection rates in an Ultimatum Game (García-Gallego et al., 2012). According to Croson and Gneezy (2009), gender differences in social decision making exist because women are more sensitive to the social cues when faced with a decision and therefore are more affected by variations in experimental setups than men, while men display relative stable behavior. Yet, our study shows that men can be equally sensitive to contextual cues. A similar observation was reported by Espinosa and Kovářík (2015), after comparing six studies investigating gender differences in prosocial behavior. The authors conclude that both male and female decision-making is sensitive to experimental variations, with males being more sensitive to self-reflection, and female behavior to prosocial framing. We build on their observations by proposing that both male and female behavior can be sensitive to social cues and framing, albeit to different aspects within the construct of interdependence.

Future experimental setups manipulating the emphasis on *dyadic* relationships may result primarily in variation in female behavior, whereas manipulating *collective* aspects may result in variation in male behavior. For example, the study by Solnick (2001) suggested that women had equal or even greater minimum acceptable offers, implying that they were more likely to reject than men. In this study players indicated a minimum acceptable offer in advance (the so-called strategy method) and did not directly interact with the other player. In this method, the impact of the decision on the other player is only indirect, thereby putting less emphasis on the relationship with the other player. This may result in a relatively higher rejection rate in females. In contrast, the study by Eckel and Grossman (2001) not only used the direct-response method, but also had four players seated across four other players, and arranged random pairings between players from each set. This setup plausibly not only led to an increased the awareness of the effect of the decision on the other player, but possibly also created a group identity among the four proposers vs. the four responders. In this study, rejection rate was higher among men.

Finally, several limitations should be considered. First, the studies did not include a qualitative measure that provided insight into the reasoning behind participants' choice to accept or reject. We were therefore only able to draw *post-hoc* inferences of why each gender responded differently to the primes. However, a long history of psychological research has established social dilemmas have high external validity, and are a preferred choice of measurement as they measure actual behavior rather than behavioral intentions (Balliet et al., 2011). Theoretical frameworks like the appropriateness framework (e.g., Weber et al., 2004) or interdependence theory (e.g., Rusbult and Van Lange, 2008) assume social decision-making is the result of a complex line of implicit reasoning resulting from multiple factors including identity, situational constraints, and the perceived relationship between players. Asking participants' to explain their reasons to accept or reject does not capture the subtle and often subconscious reasoning to make a certain choice. Furthermore, their provided explanation is likely to follow as a *post-hoc* rationalization of their choice, rather than an a priori reasoning to accept or reject. Second, Studies 1 and 2 did not include a manipulation check to control for what was on participants' mind. The reason not to include a manipulation check in Study 1 was twofold: the similarities and differences prime has been successfully used to prime self-construal for the last two decades, with a well-established effect size (Oyserman and Lee, 2008); and the content of the provided descriptions of similarities and differences allowed us to check the effect of the prime. We did not include a manipulation check in Study 2, but the fact that the manipulation check in Study 3 using the same prime had the expected results supports the effectiveness of the prime in Study 2.

## Conclusion

The current studies set out to investigate the effect of self-construal on social decision-making. Our results indicate that bringing independence or interdependence to mind has distinct downstream consequences on behavior in de Ultimatum Game, which are moderated by gender. Specifically, we find that priming the relational vs. the collective aspect of interdependence affects female and male behavior respectively. Females are more responsive to a prime emphasizing dyadic relationships, resulting in a lower rejection rate when thinking of the similarities rather than the differences with close others. Male behavior on the other hand appeared more responsive to a collective prime; rejecting more when the group norm prescribes to act in a prosocial way.

In sum, both female and male decision-making is sensitive to social cues; relationship cues influencing female behavior and collective cues influencing male behavior. Future studies may usefully include both types of manipulations, to capture the full scope of social decision-making differences between gender; either by framing within the experiment (Maddux and Brewer, 2005; Van Vugt et al., 2007) or as in the current studies, by an experimentally induced mindset.

## Ethics statement

Vaste Commissie Wetenschap en Ethiek (VCWE) All subjects were informed about the experimental procedure, and provided written consent before participating. No vulnerable populations were involved.

## Author contributions

NF, LK, and SN designed the study. NF executed the study and analyzed the data. All authors discussed the results and reviewed articles. NF and LK wrote the paper, while all authors provided input on the content and style of the manuscript. All authors gave final approval for the version to be published.

## Funding

This work was funded by a VICI grant from the Netherlands Organization for Scientific Research (453-11-005, Krabbendam) and Executive Research Agency for the European Union (Marie Curie IOF grant 302795, Novin).

### Conflict of interest statement

The authors declare that the research was conducted in the absence of any commercial or financial relationships that could be construed as a potential conflict of interest.

## Footnotes

^1^Due to the small size and diversity of the groups it was not possible to take race/ethnicity into account in the analyses.

^2^We took these questions into account in our analyses, but no effects were found.

^3^Responses were coded binary similar to Lee and Jeyaraj (2014), so there was no difference between using the independent or interdependent responses.
